# Sirolimus Induced Phosphaturia is Not Caused by Inhibition of Renal Apical Sodium Phosphate Cotransporters

**DOI:** 10.1371/journal.pone.0039229

**Published:** 2012-07-30

**Authors:** Maria Haller, Stefan Amatschek, Julia Wilflingseder, Alexander Kainz, Bernd Bielesz, Ivana Pavik, Andreas Serra, Nilufar Mohebbi, Jürg Biber, Carsten A. Wagner, Rainer Oberbauer

**Affiliations:** 1 Department of Nephrology, Medical University of Vienna, Vienna, Austria; 2 Institute of Physiology, Zurich Center for Integrative Human Physiology (ZIHP), University of Zurich, Zurich, Switzerland; 3 Department of Nephrology and Transplantation, KH Elisabethinen Linz, Linz, Austria; 4 Austrian Dialysis and Transplant Registry, Linz, Austria; Mario Negri Institute for Pharmacological Research and Azienda Ospedaliera Ospedali Riuniti di Bergamo, Italy

## Abstract

The vast majority of glomerular filtrated phosphate is reabsorbed in the proximal tubule. Posttransplant phosphaturia is common and aggravated by sirolimus immunosuppression. The cause of sirolimus induced phosphaturia however remains elusive. Male Wistar rats received sirolimus or vehicle for 2 or 7 days (1.5mg/kg). The urine phosphate/creatinine ratio was higher and serum phosphate was lower in sirolimus treated rats, fractional excretion of phosphate was elevated and renal tubular phosphate reabsorption was reduced suggesting a renal cause for hypophosphatemia. PTH was lower in sirolimus treated rats. FGF 23 levels were unchanged at day 2 but lower in sirolimus treated rats after 7 days. Brush border membrane vesicle phosphate uptake was not altered in sirolimus treated groups or by direct incubation with sirolimus. mRNA, protein abundance, and subcellular transporter distribution of NaPi-IIa, Pit-2 and NHE3 were not different between groups but NaPi-IIc mRNA expression was lower at day 7. Transcriptome analyses revealed candidate genes that could be involved in the phosphaturic response. Sirolimus caused a selective renal phosphate leakage, which was not mediated by NaPi-IIa or NaPi-IIc regulation or localization. We hypothesize that another mechanism such as a basolateral phosphate transporter may be responsible for the sirolimus induced phosphaturia.

## Introduction

Inorganic phosphate (Pi) is an essential nutrient involved in various life-sustaining processes such as cell metabolism and skeletal mineralization. The kidney is the major regulator of extracellular phosphate homeostasis. Phosphate is freely filtered in the glomerulus and mostly reabsorbed along the proximal tubule (PT) according to the organisms' needs to maintain a balanced serum phosphate. To date, three distinct sodium dependent phosphate cotransporter with a pivotal role for phosphate reabsorption in the kidney have been identified in the brush border membrane (BBM) of proximal tubule cells: NaPi-IIa (SLC34A1), NaPi-IIc (SLC343) and Pit-2 (SLC20A2) [1]–[6]. These transporters are regulated by a variety of factors and hormones including parathyroid hormone (PTH), 1,25-(OH)_2_-vitamin D_3_, and fibroblast growth factor 23 (FGF23) [4]. In the context of renal transplantation phosphate homeostasis is often disturbed and severe hypophosphatemia is a common and potential life-threatening problem during the first weeks after engraftment [7]. The preceding hyperparathyroidism and delayed reduction in FGF23 levels in patients suffering from chronic kidney disease can only partially explain reduced serum phosphate levels after kidney transplantation [8]–[10]. To date it is not fully understood to which extent immunosuppressive regimens further contribute to post-transplant hypophosphatemia. In fact several reports demonstrate the influence of various immunosuppressants including glucocorticoids, cyclosporine and tacrolimus on phosphate reabsorption in vivo [11]–[13]. Moreover, we have recently observed an aggravated and prolonged renal phosphate wasting after renal transplantation in recipients receiving sirolimus-based immunosuppression compared to patients on sirolimus-free immunosuppression [14].

Sirolimus is a potent inhibitor of the mammalian target of rapamycin (mTOR) and routinely used after solid organ transplantation to prevent rejection. mTOR has long been known for its pivotal role in regulating cell proliferation and cell growth. More recently it has been shown in the heterologous Xenopus oocyte expression system to be involved in the regulation of various solute carrier such as the creatinine transporter SLC6A8 and the renal and intestinal sodium dependent phosphate cotransporters NaPi-IIa (SLC334A1) and NaPi-IIb (SLC34A2) [15]–[17]. Furthermore it has been demonstrated that the stimulating effect of mTOR on NaPi-IIa and NaPi-IIb is suppressed by sirolimus [16], [17]. However, detailed mechanisms how sirolimus might affect renal phosphate reabsorption remain unidentified until today. The present study aimed to elucidate the underlying mechanisms involved in sirolimus induced renal phosphate loss. We hypothesized that sirolimus suppresses sodium dependent phosphate reabsorption in the PT and therefore tested for a potential role of sirolimus in the regulation of renal phosphate transport across the PT mediated by NaPi-IIa, NaPi-IIc, and Pit-2.

**Table 1:** Blood and urine parameters from vehicle and sirolimus-treated rats after two and seven days of treatment.

	2d		7d	
	Vehicle	Sirolimus	Vehicle	Sirolimus
Body weight at start (g)	245.5±11.5	249.4±13.3	191.3±3.8	184.1±3.4
Body weight after treatment (g)	225.4±6.7	215.6±10.2	238.2±5.9	195.7±5.1†
**Blood**				
pH	7.41±0.01	7.45±0.01	7.43±0.006	7.42±0.01
PCO_2_ (mmHg)	37.2±1.6	38.8±1.5	36.2±1.0	37.6±1.7
HCO_3_− (mmol/l)	24.3±0.8	26.5±0.6	24.8±2.2	24.7±0.6
Na^+^ (mmol/l)	142.0±1.9	143.2±1.2	140.7±0.9	140.7±0.8
K^+^ (mmol/l)	4.6±0.2	3.4±0.06‡	4.3±0.3	3.4±0.2
Cl^−^ (mmol/l)	96±1.5	98±3.6	94.3±3.0	93.2±1.9
Calcium (mmol/l)	2.6±0.07	2.6±0.03	2.6±0.02	2.5±0.04
Phosphate (mmol/l)	3.3±0.09	2.8±0.05†	3.0±0.08	2.4±0.09‡
Creatinine (µmol/l)	17.7±0.1	19.2±1.5	22.1±3.0	17.7±0.1
Glucose (mmol/l)	10.0±0.7	10.1±0.7	8.9±0.3	15.3±1.5†
Sirolimus trough level (µg/l)	0	20.9±2.8	0	19.6±4.5
**Urine**				
24-h Urine/body weight (ml/g)	0.03±0.002	0.06±0.006*	0.03±0.004	0.12±0.03*
pH	6.14±0.06	6.20±0.06	6.34±0.05	6.10±0.09*
Osmolarity (mOsmol/kg)	1325±340	1584±313	1189±217	1675±250*
Creatinine Clearance (ml/min)	2.3±0.1	2.1±0.2	2.1±0.3	1.9±0.2
P^−^/Creatinine (mmol/l)/(mmol/l)	12.4±0.6	17.8±0.7‡	10.4±1.1	15.8±0.9†
TmP/GFR (mmol/l)	3.0±0.1	2.5±0.06‡	2.8±0.06	2.1±0.09‡
Na^+^/Creatinine (mmol/l)/(mmol/l)	13.2±2.3	18.6±4.4*	15.7±1.1	21.5±4.2*
K^+^/Creatinine (mmol/l)/(mmol/l)	49.7±6.6	51.8±3.0	48.7±2.8	48.7±4.2
Cl^−^/Creatinine (mmol/l)/(mmol/l)	29.2±4.1	33.2±2.7	29.2±2.7	39.6±5.9†
Mg^2+^/Creatinine (mmol/l)/(mmol/l)	3.0±1.3	4.8±0.8*	3.6±1.2	4.9±0.4*
Ca^2+^/Creatinine (mmol/l)/(mmol/l)	0.4±0.1	1.0±0.4†	0.5±0.1	1.7±0.8*
HCO_3_^−^/Creatinine (mmol/l)/(mmol/l)	0.2±0.05	0.29±0.05	0.85±0.4	1.69±0.4

Values are means ± SE, n = 6/group. A summary of blood and urine parameters from rats treated with vehicle and sirolimus for two and seven days is shown. *p<0.05. †p<0.01. ‡p<0.001.

## Material And Methods

### Animals

Male Wistar rats (120–150 g, Charles River, Germany) were randomly divided into four groups: groups 1 and 3 received vehicle, groups 2 and 4 sirolimus. Each group consisted of six animals and we used samples from each rat for all experiments. Daily subcutaneous injections of sirolimus (1.5 mg/kg body weight) were given for either 2 days (group 2) or seven days (group 4), whereas control animals received daily injections of vehicle for either two days (group 1) or seven days (group 3). Sirolimus (Sigma Aldrich, Germany) was dissolved in polyethylene glycol 400, 10% polysorbat 80 and 20% dimethylacetamid. Vehicle consisted of the three solvents. Rats were maintained on a standard rodent chow containing 0.8% phosphate (Kliba AG, Kaiseraugst, Switzerland) and had access to drinking water ad libitum. All animals were placed in individual metabolic cages for three and eight days, respectively, allowing a 24 hour adaption period to the metabolic cage environment. Food and water consumption, body weight, stool and urine output were monitored daily. Urine samples were collected daily under mineral oil. All animal experiments were performed according to national and international guidelines and laws of animal welfare. Protocols were approved by the local veterinary authorities (Veterinaeramt Zurich 11/2010, Bundesministerium für Wissenschaft und Forschung Österreich 66009/4-II/10b/2010).

**Figure 1:**
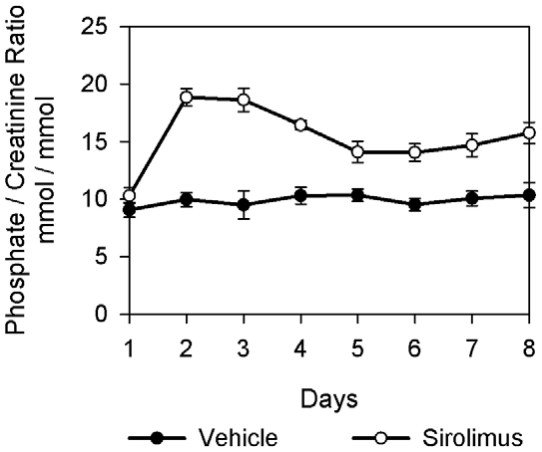
Effect of sirolimus on renal phosphate excretion. Trajectories of urinary phosphate/creatinine ratios of sirolimus and vehicle treated rats. Sirolimus caused statistically significant increased urinary phosphate excretion 24 hours after the first sirolimus injection (day 2) until after seven sirolimus injection (day 8, n = 6 for each group).

**Figure 2:**
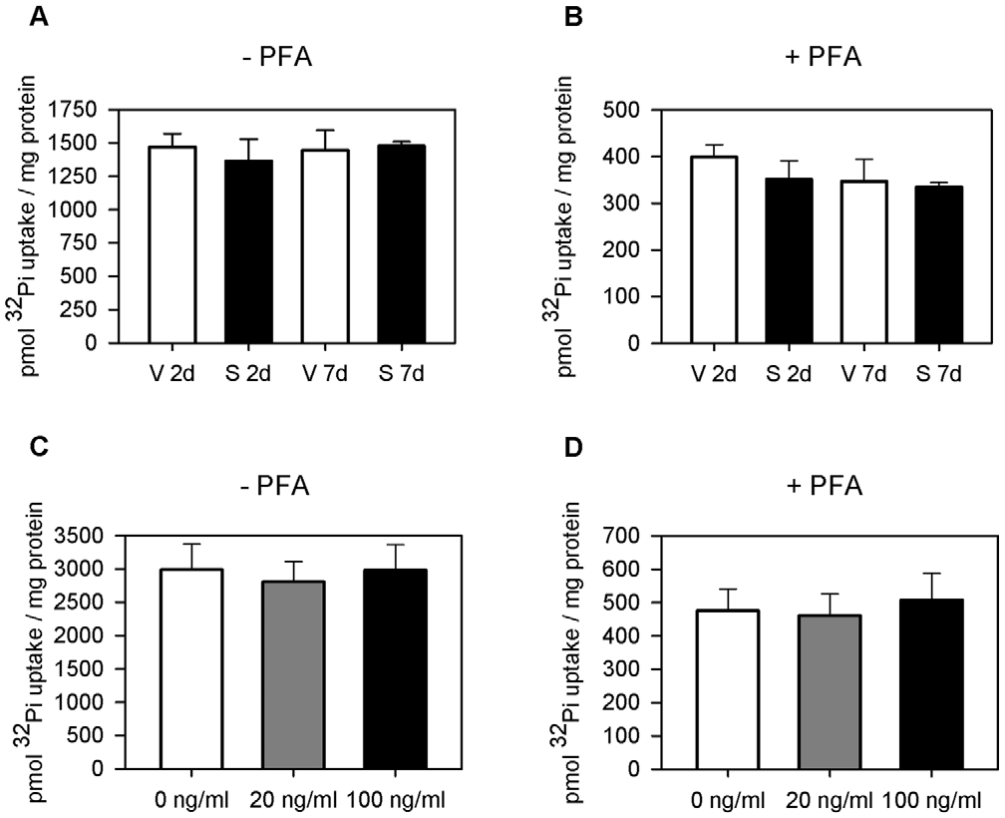
Sirolimus has no effect on apical renal phosphate transport. Effect of Sirolimus treatment for two and seven days on BBM sodium-dependent phosphate uptake in absence and presence of phosphonoformic acid (6 mM, PFA), an inhibitor of SLC34 phosphate cotransporter. Sirolimus did not alter ^32^Pi-uptake in absence (**a**) or presence (**b**) of PFA after two and seven days of sirolimus treatment (n = 6 each group). Incubation of BBMV from untreated rats with 20ng/ml and 100ng/ml sirolimus did not change Na^+^-dependent phosphate fluxes in the absence (**c**) or presence (**d**) of PFA.

**Table 2:** Serum values of phosphate regulatory hormones from vehicle and sirolimus-treated rats after two and seven days of treatment.

	2d			7d	
	Vehicle		Sirolimus	Vehicle	Sirolimus
PTH (pg/ml)	402.9±37.0	177.1±35.2†		333.9±43.6	192.5±30.5*
FGF23 (pg/ml)	259.0±15.4	267.3±21.0		249.3±19.7	144.2±12.2‡
Klotho (pg/ml)	466.5±134.6	592.6±164.5		1110.8±264.1	809.1±177.3
1,25 Dihydroxycholecalciferol (pg/ml)	123.0±10.5	154.5±9.1		130.5±12.2	177.6±19.9

Values are means ± SE, n = 6/group. A summary of blood and urine parameters from rats treated with vehicle and sirolimus for two and seven days is shown. *p<0.05. †p<0.01. ‡p<0.001.

### Blood and Urine Analysis

At the end of the experiments rats were anesthetized by inhalation of Isoflurane/air and heparinized arterial blood was collected from the tail artery and immediately analyzed for pH, blood gases, and electrolytes on a Radiometer ABL 800 Flex blood gas analyzer (Radiometer, Copenhagen, Denmark). Serum creatinine, serum phosphate and serum glucose concentrations were determined using the clinical chemistry analyzer Piccolo Xpress (LabForce, Nunningen, Switzerland). Vitamin D plasma levels were determined using the 1,25 Dihydroxy Vitamin D RIA (Immunodiagnostic Systems Ltd., Baldon, UK) according to the manufacture's protocol. The levels of intact fibroblast growth factor 23 (intact FGF23, Kainos Laboratories Inc., Tokyo, Japan) intact parathyroid hormone (intact PTH, Immunotopics Inc., San Clemente CA, USA) and soluble klotho (Immuno-Biological Labratories Co., Minneapolis, USA) were measured by a two-site enzyme-linked immunosorbent assay in rodent serum samples, according to the manufacturer's protocol. Inter-assay and intra-assay coefficients of variation were below 5% for all laboratory analyses. Creatinine clearance was calculated from the measured values. Sirolimus blood levels were measured using HPLC-mass spectrometry [18]. For urinary pH, PCO_2_ and calculated HCO_3_^−^ measurement urine was aspirated from the collectors (urine was collected under mineral oil) into syringes and injected into the blood gas analyzer. Urinary creatinine was analyzed using the Jaffé method [19]. Urinary phosphate was determined by endpoint method with sample blanking [19], [20]. Urinary K^+^, Na^+^, Cl,^−^ Ca^2+^ and Mg^2+^ concentrations were measured using a chemistry analyzer (Cobas Integra 800, Roche, Urinary osmolarity was determined using a One-Ten Osmometer (Fiske Associates, Norwood, Massachussettes, USA) by examination of freezing-point depression. A test for low molecular weight proteinuria was performed. Urine samples were normalized for creatinine and samples containing 10 mg creatinine were then solubilized in Laemmli sample buffer and SDS-PAGE was performed on a 15% polyacrylamid gel. Colloidal coomassie blue staining was performed for 1h at room temperature. After destaining for 2h, the gel was photographed and dried. Five micrograms of BSA served as a positive control.

**Figure 3:**
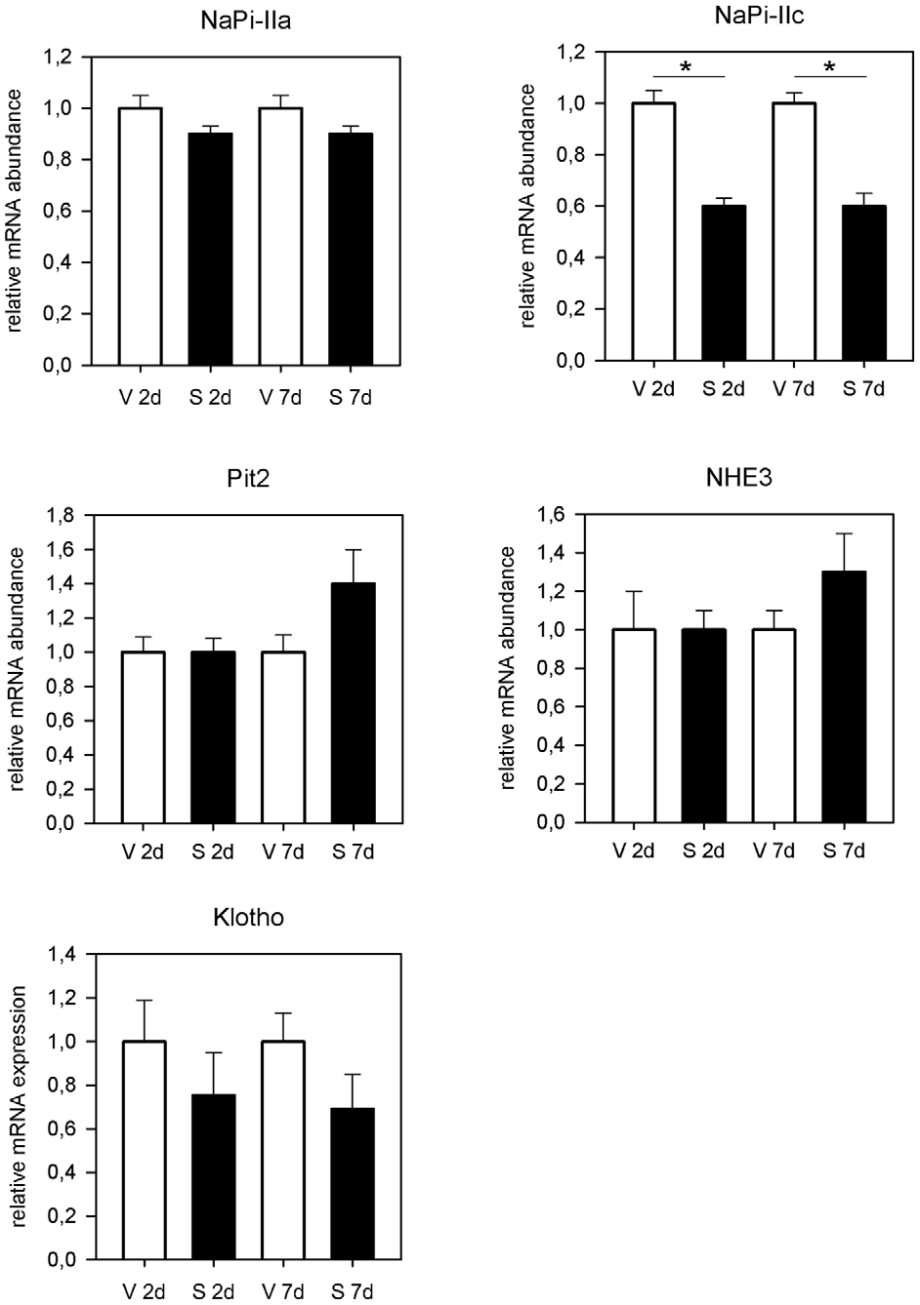
Sirolimus has no effect on renal phosphate transporter mRNA abundance. Results of the semiquantitative RT-qPCR for NaPi-IIa, NaPi-IIc, Pit-2, NHE3 and klotho after two and seven days of sirolimus treatment. mRNA abundance for NaPi-IIa, klotho, Pit2 and NHE3 was not different between groups after two and seven days of sirolimus treatment. NaPi-IIc mRNA abundance was significantly lower in sirolimus treated animals after two and seven days. * p<0,05.

**Figure 4:**
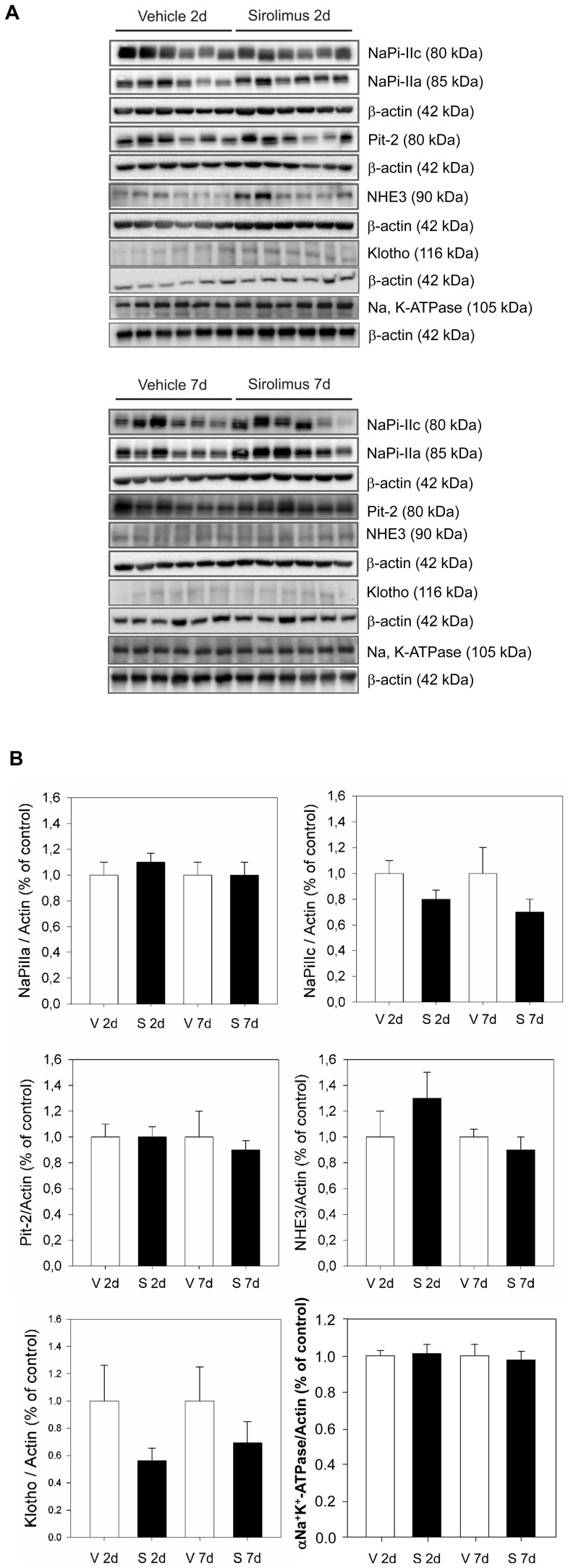
Sirolimus does not alter renal phosphate transporters. Sirolimus treatment for two and seven days does not alter protein expression levels of NaPi-IIa, NaPi-IIc, Pit-2, klotho and NHE3 in the brush border membrane. Brush border membranes or total membrane protein were prepared from kidneys of sirolimus and vehicle injected rats (n = 6) and 10 µg of brush border membranes or 35 µg of total membrane protein were loaded per lane for immunoblotting. **a** Membranes were tested for NaPi-IIa, NaPi-IIc, Pit-2, NHE3, and the αNa+/K^+^-ATPase subunit and stripped for reprobing with ß-actin to control for loading. **b** Densitometric analysis of all immunoblots with the appropriate software was performed and bands of the proteins of interest were normalized against ß-actin and the respective vehicle groups. Sirolimus treatment for two and seven days did not change the abundance of NaPi-IIa, NaPi-IIc, Pit-2, klotho and NHE3 compared to vehicle.

**Figure 5:**
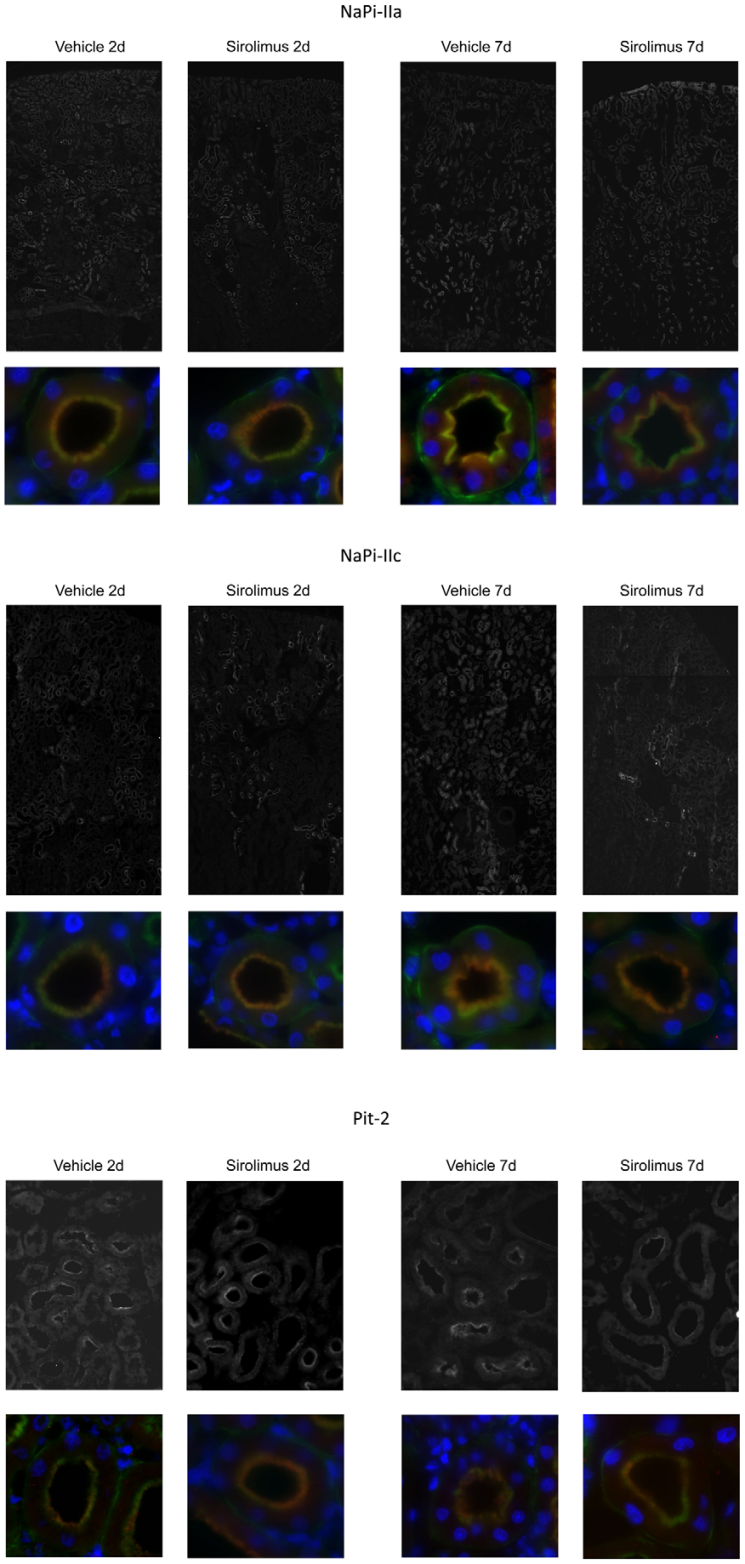
Sirolimus has no effect on localization of renal phosphate transporters. Effect of sirolimus treatment for two and seven days on NaPi-IIa, NaPi-IIc and Pit-2 localization. NaPi-IIa, NaPi-IIc or Pit2 staining (red) was observed in the BBM of early proximal tubules, and colocalized with β-actin as a marker of the BBM (green) as indicated by the yellow overlay. Nuclei were stained with DAPI (blue). No difference was observed between animals treated with vehicle or sirolimus for either 2 or 7 days (n = 5 per group). Original magnification 630 x.

### RNA extraction and semi-quantitative real time qRT-PCR

Total RNA from kidneys was extracted using the Qiagen RNeasy Mini Kit (Qiagen, Hombrechtikon, Switzerland). Snap-frozen kidneys were homogenized in RLT buffer (Qiagen) supplemented with ß-mercaptoethanol to a final concentration of 1%. Subsequently, 200 µl of each homogenate were used for total RNA isolation according to the manufacturer's protocol. DNAse digestion was performed using the RNase-free DNAase set (Qiagen, Hilden, Germany). Quality and concentration of the isolated RNA preparations were analyzed spectroscopically using the 2100 Bioanalyzer (Agilent Technologies) and the NanoDrop ND-1000 spectrophotometer (NanoDrop Technologies). RNA samples were diluted to a final concentration of 100 ng/µl and 3 µl were used for cDNA preparation using the TaqMan Reverse Transcriptase Reagent Kit (Applied Biosystems/Roche, Foster City, CA). Primers and probes (Microsynth, Balgach, Switzerland) of all genes of interest were designed using Primer Express Software (v.2.0.; Applied Biosystems), and primers were tested by PCR with kidney cDNA and always resulted in a single product of the expected size (data not shown). Sequences of primers and probes are listed in Data S1. Real-time PCR reactions were performed using the TaqMan Universal PCR Master Mix (Applied Biosystems). Briefly, 3 µl cDNA, 0.8 µl of each primer (25 µM) and 0.4 µl of labeled probe (5 µM), 5 µl RNase-free water, 1 0µl TaqMan Universal PCR Master Mix added to a final volume of 20 µl. Reactions were run in 96-well optical reaction plates using the Prism 7500 fast Real-Time PCR cycler. Cycling conditions were set to one cycle for 2 min at 50°C, 10 min at 95°C, followed by 40 cycles at 95°C (10s) and 60°C (1 min) with auto ramp time. All reactions were run in triplicate, and one negative control without addition of the multiscribe reverse transcription enzyme was included for each sample. The relative abundance of target mRNA was calculated to a reference mRNA (hypoxanthine-guanine-phosphoribosyltransferase; HPRT). Relative expression levels were calculated as R = 2^[Ct(HPRT) _ Ct(gene)]^, where Ct is the cycle number at which the fluorescence intensity is above background levels (threshold).

### Brush border membrane vesicle preparation and phosphate uptake experiments

Frozen kidneys were used to prepare total membrane proteins and brush border membrane vesicle (BBMV) using the Mg^2+^ precipitation technique as previously described [21], [22]. The phosphate transport rate into BBMV was measured in freshly prepared BBMV at 25°C in the presence of inward gradients of 100 mM NaCl or 100 mM KCl and 0.1 mM K-phosphate. The substrate Pi was made with 0.125 mM K2HPO4 and 32P (1μCi/ml) to give a final concentration 0.1 mM close to the expected apparent Km for Pi for Na+-dependent transport in renal BBMV. The stop solution contained 100 mM Mannitol, 5 mM Tris–HCl pH 7.4, 150 mM NaCl, 5 mM Pi. Na+-dependence was established by incubating BBMV in solutions in which KCl replaced NaCl equimolarly. Phosphate uptake was determined after 60 s, representing initial linear conditions, and after 120 min, to determine the equilibrium values. In order to distinguish between Na+-dependent Pi uptake mediated by SLC34 family members (e.g., NaPi-IIa and NaPi-IIc) and other Na+-dependent phosphate transporters such as SLC20 family members (e.g., Pit-1 and Pit-2), we used trisodium phosphonoformic acid (PFA, final concentration 6 mM) added to the same solution with 107mM NaCl. PFA is known to be an inhibitor of BBMV phosphate uptake since decades [23], [24] but has previously been shown to have a higher selectivity for type II (i.e. NaPi-IIa and NaPi-IIc, SLC34) than type III (i.e. Pit-2, SLC20) phosphate transporters at these concentrations [2], [25]–[27]. Total protein concentration was measured using the Bio-Rad Protein Assay kit, Bio-Rad, Hercules, CA, USA. BBMV were stored at −80°C until further use.

### Immunoblotting

Ten micrograms of renal BBM protein or 35µg of total membrane protein was solubilized in Laemmli sample buffer and SDS-PAGE was performed on 8% polyacrylamid gels. Proteins were transferred electrophoretically from gels to polyvinylidene difluoride membranes (Immobilon-P, Millipore, Bedford, MA). After blocking with 5% milk powder in tris-buffered saline containing 0.1% tween 20 for 60min, the blots were incubated with the respective primary antibodies: rabbit polyclonal anti-NaPi-IIa (1∶6,000) [28], rabbit polyclonal anti-NaPi-IIc (1∶10,000) [29], rabbit polyclonal anti-Pit-2 (1∶3,000) (kindly provided by V. Sorribas, University of Zaragosa, Spain), rabbit polyclonal anti NHE3 (1∶5,000)(generated by immunization of rabbits with a peptide linked to KLH, Pineda antibody service, Berlin, Germany) rabbit polyclonal anti-klotho (Abcam, Cambridge, UK) and mouse monoclonal anti-β-actin antibody (42kD; Sigma, St. Louis, MO; 1∶5,000) overnight at 4°C. After washing and subsequent blocking, blots were incubated with the secondary antibody (donkey anti-rabbit or sheep anti-mouse antibodies linked to horseradish peroxidase 1∶10,000, (GE Healthcare, Little Chalfont, Buckinghamshire, UK) or goat anti-rabbit antibody 1∶5,000 linked to alkaline phosphatase (Promega, Madison, WI) for 1h at room temperature. Antibody binding was detected with the Immobilon western chemiluminescence kit (Millipore, Billerica, MA), using the DIANA III-chemiluminescence detection system (Raytest, Straubenhardt, Germany). All images were analyzed using appropriate software (Advanced Image Data Analyzer, Raytest) to calculate the protein of interest/ß-actin ratio.

### Immunohistochemistry

Anesthetized rats were fixed by vascular perfusion through the left ventricle. The thorax was opened and the fixative was manually injected through a 50 ml syringe. The fixative consisted of 3% paraformaldehyde, 0.05% picric acid in 0.1M cacodylate buffer (pH 7.4; containing 3 mM MgCl_2_ and adjusted to 300 mosmol/l with sucrose) and 4% hydroxyethyl starch in saline (HAES sterile; Fresenius, Stans, Switzerland). After ten minutes the fixative was washed out by perfusion with PBS. Kidneys were then removed and stored in PBS overnight at 4°C. Coronal slices of fixed kidneys were then frozen in liquid propane and cooled with liquid nitrogen and stored −80°C. Serial sections, 5 μm thick, were cut at −20°C on a cryomicrotome (CM 1850-1-1, Leica Microsystems, Nussloch, Germany) mounted on thermo scientific superfrost plus glass slides (Thermo Fischer Scientific Inc, Braunschweig, Germany), thawed, and kept in cold PBS until further processing for staining. Before immunofluorescence staining, sections were pretreated with blocking solution (Normal Goat Serum 10% in PBS with 0.5% bovine serum albumin,0.04%Na-Azide) for 60 min at room temperature. After blocking sections were incubated with the primary antibody overnight at 4°C either with a rabbit anti-rat antiserum against the NaPi-IIa protein [28] diluted 1∶1,000 or with an immunopurified rabbit anti-mouse NaPi-IIc [29] diluted 1∶1,500, or a rabbit anti-rat Pit-2 [2] diluted 1∶250. All primary antibodies were diluted in PBS with 0.5% bovine serum albumin. Sections were then rinsed three times with PBS and covered for 90 minutes at room temperature with Alexa Fluor 555 goat–anti-rabbit IgG (1∶1,000, Invitrogen), FITC–phalloidin (Molecular probes, Eugene, OR, USA, 1∶200), and 4,6-diamidino-2-phenylindole (DAPI; Sigma, St Louis, MO) diluted 1∶500. Finally, the sections were rinsed three times with PBS, cover slipped using DAKO-Glycergel (Dakopatts) containing 2.5% 1,4-diazabicyclo [2.2.2] octane (Sigma) as a fading retardant. Immunohistochemistry images were acquired with a Leica DFC490 charged-coupled device camera attached to a Leica DM 6000 fluorescence microscope (Leica, Wetzlar, Germany) using equivalent camera parameters for kidneys sections stained with the same primary antibody. Pictures were processed using Adobe Photoshop (overlays).

### Statistical analysis

All data are summarized as mean ± SE and were analyzed using the unpaired Student's t- test with p values ≤0.05 considered as statistically significant. Urinary phosphate/creatinine ratio course throughout the experiment was analyzed using a mixed linear model. We calculated the correlation coefficients for magnesium, calcium, sodium, chloride and potassium in correlation to phosphate in urine. SAS V9.2 for windows was used as statistical software (2008 SAS System Inc., Cary, NC, USA).

## Results

### Animal model, blood and urine parameter

Mean body weight was similar in all groups at the beginning of the experiment and after two days of treatment with sirolimus but was significantly lower in sirolimus treated animals after seven days of treatment (238.2 g ±5.9 g vs 195.7 g ±5.1 g, p = 0.0003) (table 1). Sirolimus treatment with 1.5 mg/kg/body weight for two and seven days resulted in plasma trough levels (µg/l) of 20.9±2.8 at day 2 and 19.6±4.5 at day seven. The treatment with the mTOR inhibitor did not change acid-base homeostasis as determined by arterial blood gas and urine analysis. Blood glucose levels did not differ between groups after two days but sirolimus treated animals exhibited higher serum glucose levels at day seven. Sirolimus treatment for two and seven days had no effect on creatinine clearance and serum creatinine levels. Sirolimus treatment was associated with polyuria indicated by a significantly higher 24-h urine/body weight (ml/g) after two and seven days (0.03±0.002 vs 0.06±0.006, p = 0.004, and 0.03±0.004 vs 0.12±0.03 p = 0.03, respectively). Accordingly, urine osmolarity (mOsmol/kg) was significantly decreased in sirolimus treated animals after seven days but was not significantly decreased after two days (1325±340 vs 1584±313, p = 0.2 and 1189±217 vs 1675±250 p = 0.02, respectively). Serum Na^+^, Cl^−^, Ca^2+^ showed no significant changes, however, serum K^+^ was significantly lowered in sirolimus treated rats after two days (4.6±0.2 vs 3.4±0.06, p = 0.0001). Urinary Na^+^/creatinine ratios ((mmol/l)/(mmol/l)) were significantly higher after two and seven days treatment (13.2±2.3 vs 18.6±4.4, p = 0.02 and 15.7±1.1 vs 21.5±4.2, p = 0.02). Cl^−^/Creatinine ratio ((mmol/l)/(mmol/l)) was significantly higher after seven days (29.2±2.7 vs 39.6±5.9, p = 0.003) but was not different after two days. Mg^2+^/Creatinine ratios and Ca^2+^/Creatinine ratios ((mmol/l)/(mmol/l)) were significantly higher after two and seven days (magnesium 3.0±1.3 vs 4.8±0.8, p = 0.02 and 3.6±1.2 vs 4.9±0.4, p = 0.04; calcium 0.4±0.1 vs 1.0±0.4, p = 0.006 and 0.5±0.1 vs 1.7±0.8, p = 0.01). Only very weak overall correlations of urinary phosphate with magnesium, sodium and chloride, calcium and potassium were found (Data S1). Whereas sirolimus treatment for two days did not change urinary pH, animals treated with sirolimus for seven days excreted a slightly more acidic urine compared to vehicles (6.34±0.05 vs 6.10±0.09, p = 0.03). However, the bicarbonate excretion into the urine remained unchanged between groups as determined by HCO_3_^−^/creatinine ratio. Urinary protein excretion was similar between all groups excluding proteinuria (data not shown). Urinary glucose/creatinine ratio ((mmol/l)/(mmol/l)) was significantly higher only after seven days (0.3±0.1 vs. 135.4±113.6, p = 0.03).

### Functional studies of phosphate homeostasis

Urine phosphate/creatinine ratio was higher in sirolimus treated rats beginning at 24 hours after the first injection and continued to be higher throughout the whole experiment for two and seven days (figure 1, p<0.0001) Furthermore, serum phosphate (mmol/l)) was significantly lower in sirolimus treated rats after two and seven days (3.3±0.09 vs 2.8±0.05, p<0.01 and 3.0±0.08 vs 2.4±0.09, p<0.001) (table 1). Moreover Tmp/GFR (mmol/l) was significantly lower in sirolimus treated rats after two and seven days (3.0±0.1 vs 2.5±0.06, p<0.001 and 2.8±0.06 vs 2.1±0.09, p<0.001, respectively). While serum-PTH (pg/ml) was lower in sirolimus treated rats after two and seven days (403±37 vs 177±35, p = 0.001 and 334±44 vs 193±31, p = 0.02), FGF 23 levels (pg/ml) were unchanged after two days but significantly lower in sirolimus treated rats after seven days (259±15 vs 267±21 p>0.05 and 249±20 vs 144±12, p = 0.001). Soluble klotho serum levels and vitamin D_3_ serum levels were not significantly affected by sirolimus treatment (table 2). BBMV ^32^Pi uptakes in the absence and presence of phosphonoformic acid (PFA) were similar in sirolimus and vehicle injected rats after two and seven days and revealed no different characteristics in phosphate influx (figure 2). Moreover, incubation of BBMV from untreated rats with two different sirolimus concentrations, 20ng/ml and 100ng/ml respectively, during the preparation and uptake procedure had no effect on Na^+^-dependent phosphate fluxes (figure 2).

### Renal phosphate regulation on the transcriptional level

Results from semiquantitative RT-qPCR for NaPi-IIa, NaPi-IIc and Pit-2 revealed only minor changes in mRNA abundance for these sodium dependent phosphate cotransporters in the PT of kidneys from sirolimus treated rats (figure 3). In detail mRNA abundance for NaPi-IIa, Pit2 as well as for klotho was not different between groups after two and seven days of sirolimus treatment. NaPi-IIc mRNA abundance was significantly lower in sirolimus treated animals after two and seven days (1±0.05 vs 0.6±0.05, p = 0.0003 and 1±0.04 vs 0.6±0.0, p = 0.0002). Furthermore NHE3 mRNA levels were not affected by sirolimus treatment. Our microarray data showing no significant difference between NaPi-IIa, NaPi-IIc and NHE3 confirm these RT-qPCR results. Microarray analysis was performed on kidney samples from the same animals. In total 154 features were identified as significant differentially expressed when comparing the gene expression profiles of rat kidneys after seven days of treatment with either sirolimus or vehicle with a fold change over 1.5, resulting in 139 down-regulated and 15 up-regulated genes in the sirolimus group. According to PANTHER classification down-regulated transcripts belong to response to stimulus, metabolic processes, immune system, transport, and signal transduction. Up-regulated transcripts belong also to response to stimulus, immune system, and transport but also to blood coagulation and regulation of vasoconstriction. Interestingly transcriptome analyses revealed several potential candidate genes that may be involved in tubular phosphate transport. SLC17A4, a putative sodium-dependent phosphate transporter protein was significantly down regulated in sirolimus treated rats compared to vehicle [30].

### Renal phosphate regulation on the protein level

Sirolimus treatment for two and seven days did not lead to a significant alteration in protein expression levels as determined by immunoblotting for NaPi-IIa, NaPi-IIc, Pit-2, klotho, NHE3, and the alpha subunit of the Na^+^/K^+^-ATPase (figure 4). The relative protein abundance of NaPi-IIa, NaPi-IIc and Pit-2 remained similar between groups after two and seven days. Additionally, immunostaining for NaPi-IIa, NaPi-IIc, and Pit-2 did not reveal any difference in transporter distribution and respective localization at the apical surface of proximal tubule cells between sirolimus and vehicle treated rats after two and seven days (figure 5).

## Discussion

This study demonstrates that sirolimus causes hypophosphatemia and hyperphosphaturia as observed in patients treated with this immunosuppressant [14]. The rats receiving sirolimus also recapitulated other side effects such as hyperglycemia and glucosuria often observed under sirolimus treatment [31]. Our data indicate that the sirolimus induced urinary phosphate wasting is not caused by dysfunction of the three currently known phosphate transporters located in the BBM of the proximal tubule. Our results furthermore indicate an intact physiological feedback mechanisms of the phosphate regulating hormones PTH, 1,25 dihydroxycholecalciferol, FGF23 and its cofactor klotho to counterbalance the renal phosphate loss.

Sirolimus treated rats did not develop signs of general renal failure or a more generalized Fanconi-like dysfunction of the PT as indicated by the absence of bicarbonaturia or proteinuria. Furthermore NHE3 mRNA as well as protein abundance was not different between groups. The dose of sirolimus applied here is comparable to that used in similar animal studies [32]–[34].

Several lines of evidence support the conclusion that sirolimus did not induce phosphaturia by primarily acting on BBM phosphate transporters despite a significantly lower mRNA abundance of NaPi-IIc. i) the rates of Na^+^-dependent phosphate uptake into BBMV was not affected by treatment of rats with sirolimus or acute incubation in vitro with high concentrations of sirolimus, ii) mRNA and protein expression of NaPi-IIa and Pit-2 were unaffected by sirolimus treatment. Solely mRNA expression of NaPi-IIc was significantly down regulated after two and seven days. However, sirolimus did not alter protein expression of NaPi-IIc iii) the subcellular localization of these three transporters with predominant localization in the BBM of the early PT was not altered, iv) microarray analysis of renal transcripts did not detect changes in any mRNA related to the known phosphate transporters or proteins known to be involved in their expression or activity, and v) hormonal changes are consistent with compensatory adaptations but not with causing phosphaturia.

Our results are in apparent contradiction with two recent reports suggesting that the mTOR pathway may regulate the renal and intestinal phosphate cotransporters NaPi-IIa and NaPi-IIb [16], [17] Both studies describe the in vitro stimulation of NaPi-IIa and NaPi-IIb induced phosphate transport in Xenopus oocytes by coexpression of the mTOR kinase and the reversion of the stimulatory effect by rapamycin. In contrast, our study is performed in vivo and fails to detect any impact on the renal NaPi-IIa cotransporter. Moreover, Moz et al demonstrated that the calcineurin A beta subunit is involved in the regulation of NaPi-IIa in vivo and required for the normal adaptation of NaPi-IIa expression in response to changes in dietary phosphate intake [35]. The requirement of calcineurin A beta may explain the phosphaturia observed in calcineurin inhibitors such as tacrolimus. However, sirolimus does not directly affect calcineurin A beta.

It has been previously shown that klotho may exert some phosphaturic effects independently from FGF23 [36]. However sirolimus induced phosphaturia in our in vivo rat model did not alter renal klotho mRNA and protein expression or serum levels.

We also performed whole kidney transcript anaylasis using microarrays to detect candidate targets of sirolimus that may participate in the induction of phosphaturia. However, the analysis of significantly altered transcripts did not reveal any genes with a clear relation to renal phosphate transporters or their regulation. Microarray data indicated downregulation of SLC17A4. This transporter belongs to the SLC17 superfamily of transporters with subgroups of urate and vesicular gluatamte transporters [37]. Initially several transporters from this family have been assigned as phosphate transporters due to the induction of phosphate transport when heterologously expressed in Xenopus oocytes [38], [39]. More recent experiments identified urate or glutamate as physiological substrates [37]. However, the substrates of SLC17A3 have not been reported to date and its exact expression pattern not reported. None of the altered transcripts has been connected to renal phosphate handling to date. Further analysis of transcripts affected by sirolimus treatment is required to understand their biological significance.

The direct target(s) of sirolimus causing phosphaturia remain(s) elusive. Our results suggest that direct dysregulation of BBM phosphate transporters can be excluded and that other mechanisms must be considered. Two alternative targets might be either basolateral PT phosphate exit pathways or phosphate transport mechanisms located in the more distal nephron. Earlier studies suggested the presence of phosphate absorbing mechanisms in the distal tubule, however, their functional significance, regulation, molecular identity, or even existence have remained elusive to date [40], [41]. Completion of phosphate absorption in the PT requires (a) exit pathway(s) across the basolateral membrane. Only few data exist analyzing functional properties of the basolateral phosphate transport mechanisms but the molecular identity of basolateral phosphate transport proteins is unknown to date [42], [43].

### Conclusion

Sirolimus induces renal phosphate wasting and the absence of changes in the expression of all known renal phosphate transporters and normal BBM phosphate transport rates suggest an alternative mechansim. Moreover, the regulation of the major known phosphate regulating hormones, PTH, 1,25 Dihydroxycholecalciferol, FGF23 in response to hypophosphatemia was intact and suggests rather compensatory adaptation. Sirolimus might affect other mechanisms that could contribute to overall renal phosphate handling such as either basolateral exit pathways for phosphate in the PT or other elusive transport pathways in downstream nephron segments. Clearly, further studies are needed to unravel the molecular mechanisms causing sirolimus induced phosphaturia.

## Supporting Information

Data S1 **Supporting Information**
(DOCX)
