# Supplementary material for: Sirolimus Induced Phosphaturia is Not Caused by Inhibition of Renal Apical Sodium Phosphate Cotransporters
Source: PLoS One. 2012 Jul 30;7(7):e39229. doi: 10.1371/journal.pone.0039229 (PMC3408497; doi:10.1371/journal.pone.0039229)
Supplement: Data S1 — Supporting Information (DOCX) [file pone.0039229.s001.docx]

**Supplemental data to**

**Sirolimus induced phosphaturia is not caused by inhibition of renal apical sodium phosphate cotransporters in rats**

Maria Haller^1,2,3^, Stefan Amatschek^1^, Julia Wilflingseder^1^, Alexander Kainz^1^, Bernd Bielesz^1^, Ivana Pavik^2^, Andreas Serra^2^, Nilufar Mohebbi^2^, Jürg Biber^2^, Carsten A. Wagner^2^, Rainer Oberbauer^1,3,4^

^1^ Department of Nephrology, Medical University of Vienna, Vienna, Austria

^2^ Institute of Physiology and Zurich Center for Integrative Human Physiology (ZIHP), University of Zurich, Zurich, Switzerland

^3^ Department of Nephrology and Transplantation, KH Elisabethinen Linz, Linz, Austria

^4^ Austrian Dialysis and Transplant Registry, Linz, Austria

**Table S1** Primers and Probes used. List of sequences of primers and probes used in these study.

| **Gene** | **Primers** | **Probe** |
| --- | --- | --- |
| **AQP2** | FW: 5'-TGGTGCTGTGCATCTTTGCCT-3' | 5'-ACCTCCTTGGGATCTATTTCACCGG-3' |
|  | RV: 5'-ACTTGCCAGTGACAACTGCTG-3' |  |
| **NaPi-Iia** | FW: 5'-GGAATCACAGTCTCATTCGGATT-3'  RV: 5'-ATGGCCTCTACCCTGGACATAG-3' | 5'-TGTCAACCAGAGACAAAAGAGGCTTCCACT -3' |
| **NaPi-Iic** | FW: 5'-GGGATCGGGATGAATTTCAGA-3'  RV: 5'-GGGCCAGCTCACTCAGTCTCT-3' | 5'-ACGGCATCTTCAACTGGCTCACAGTGTT-3' |
| **Pit-2** | FW: 5'-CCTGCTTTGGGTCCTTTGC-3'  RV: 5'-CAGCCACAGGGCCACAAG-3' | 5'-ACGGTGGCAACGATGTGAGCAATG-3' |
| **HPRT** | FW: 5'-GCTGAAGAT TTGGAAAAGGTGTTTA-3' | 5'- TTATGGACAGGACTGAAAGACTTGCTGGAGATG-3' |
|  | RV: 5'-ACACAGAGGGCCACAATGTGA- 3' |  |
| **NHE3** | FW: 5'-CACCCACCACACGTTGCA-3' | 5'-CTCTACAAGCCTCGGCAGGAGTACAA-3' |
|  | RV: 5'-GTGAGCTCGTGCCGACTGT-3' |  |
| **Klotho** | FW: 5'-TATCTCAAGAAGTTCATAATGGAAAGC-3' | 5'-TAAAAGCCATCAGGCTGGATGGGG-3' |
|  | RV: 5'-GAGGGACCATGCGGTGTA-3' |  |

**Table S2** Summary of major results.

|  | **2d** | | **7d** | |
| --- | --- | --- | --- | --- |
|  | **Vehicle** | **Sirolimus** | **Vehicle** | **Sirolimus** |
| **Functional phosphate studies** |  |  |  |  |
| Serumphosphate (mmol/l) | 3.3 ± 0.09 | 2.8 ± 0.05† | 3.0 ± 0.08 | 2.4 ± 0.09‡ |
| Urine phosphate/creatinine ratio | 12.4 ± 0.6 | 17.8 ± 0.7‡ | 10.4 ± 1.1 | 15.8 ± 0.9† |
| TmP/GFR (mmol/l) | 3.0 ± 0.1 | 2.5 ± 0.06‡ | 2.8 ± 0.06 | 2.1 ± 0.09‡ |
| BBMV phosphate uptake (pmol/mg) | 1091 ± 77 | 1030 ± 129 | 1145 ± 126 | 1158 ± 32 |
| **Phosphatonins** |  |  |  |  |
| PTH (pg/ml) | 403 ± 37 | 177 ± 35† | 334 ± 44 | 193 ± 31* |
| FGF 23 (pg/ml) | 259 ± 15 | 267 ± 21 | 249 ± 20 | 144 ± 12‡ |
| Klotho (pg/ml) | 466.5 ± 134.6 | 592.6 ± 164.5 | 1110.8 ± 264.1 | 809.1 ±177.3 |
| 1,25 Dihydroxycholecalciferol (pg/ml) | 123 ± 11 | 155 ± 9 | 131 ± 12 | 178 ± 20 |
| **Transcription** |  |  |  |  |
| qPCR NaPi IIa (2^^HPRT-NaPi IIa^) | 1 ± 0.05 | 0.9 ± 0.03 | 1 ± 0.05 | 0.9 ± 0.03 |
| qPCR NaPi IIc (2^^HPRT-NaPi IIc^) | 1 ± 0.05 | 0.6 ± 0.05‡ | 1 ± 0.04 | 0.6 ± 0.05‡ |
| qPCR Pit-2 (2^^HPRT-Pit2^) | 1 ± 0.09 | 1 ± 0.08 | 1 ± 0.1 | 1.4 ± 0.2 |
| qPCR Klotho (2^^18S-Klotho^) | 1 ± 0.19 | 0.75 ± 0.19 | 1 ± 0.13 | 0.7 ± 0.15 |
| qPCR NHE3 (2^^HPRT-NHE3^) | 1 ± 0.2 | 1 ± 0.1 | 1 ± 0.1 | 1.3 ± 0.2 |
| **Translation** |  |  |  |  |
| WB NaPi IIa (Actin/NaPi IIa Ratio) | 1 ± 0.1 | 1.1 ± 0.07 | 1 ± 0.1 | 1 ± 0.1 |
| WB NaPi IIc (Actin/NaPi IIc Ratio) | 1 ± 0.1 | 0.8 ± 0.07 | 1 ± 0.2 | 0.7 ± 0.1 |
| WB Pit-2 (Actin/Pit-2 Ratio) | 1 ± 0.1 | 1 ± 0.08 | 1 ± 0.2 | 0.9 ± 0.07 |
| WB Klotho (Actin/Klotho Ratio) | 1 ± 0.26 | 0.6 ± 0.09 | 1 ± 0.25 | 0.7 ± 0.15 |
| WB NHE3 (Actin/NHE3 Ratio) | 1 ± 0.2 | 1.3 ± 0.2 | 1 ± 0.06 | 0.9 ± 0.1 |
| IHC NaPi IIa |  | No difference |  | No difference |
| IHC NaPi IIc |  | No difference |  | No difference |
| IHC Pit-2 |  | No difference |  | No difference |

Values are means ± SE, n = 6/group. A summary of all major results from rats treated with vehicle and sirolimus for two and seven days is shown. *p < 0.05. †p < 0.01. ‡p < 0.001.

**Table S3a** Effect of Sirolimus on electrolyte concentration in the urine. P-values of mixed linear models of Glucose, Calcium, Magensium, Sodium, Chloride, Potassium and Phosphate in the Urine. Group refers to the sirolimus effect, day refers to the time effect over seven days and interaction describes effect modification. With the exception of magnesium and sodium, no adjusted group effect could be observed.

| **Parameter** | **Group** | **Day** | **Interaction Group*Day** |
| --- | --- | --- | --- |
| Glucose | 0.1365 | <.0001 | <.0001 |
| Calcium | 0.0117 | 0.0003 | <.0001 |
| Magnesium | 0.0029 | 0.0025 | 0.0007 |
| Sodium | 0.0068 | 0.0002 | 0.0130 |
| Chloride | 0.0486 | 0.1637 | 0.0044 |
| Potassium | 0.6620 | 0.0157 | 0.2097 |
| Phosphate | <.0001 | <.0001 | <.0001 |

**Table S3b** Correlation of urinary electrolyte concentration with phosphate in the urine. Correlation coefficients (corr) of calcium, magnesium, sodium, chloride and potassium (Var) to phosphate (phos) in the Urine. Only calcium showed a somewhat stronger correlation with urinary phosphate excretion.

| **Parameter** | **estimate Mean (Var)** | **estimate Mean (Phos)** | **partial corr** | **repeated measures corr** | **overall corr** |
| --- | --- | --- | --- | --- | --- |
| Calcium | 0.8312 | 11.9963 | 0.43092 | 0.78812 | 0.68137 |
| Magnesium | 4.4622 | 12.4281 | 0.29622 | 0.35792 | 0.52572 |
| Sodium | 15.9049 | 12.4935 | 0.03887 | 0.22421 | 0.37141 |
| Chloride | 32.9183 | 12.3557 | 0.12278 | 0.47265 | 0.40074 |
| Potassium | 50.8180 | 12.4281 | 0.22331 | 0.35944 | 0.10918 |

# Functional Genomics

## Methods

### Microarray hybridization and bioinformatic workflow

Kidney gene expression analysis was performed according to the NuGEN -recommended protocol using the Affymetrix GeneChip Rat Gene 1.0 ST Array containing probes for 27,342 annotated genes. Total RNA (200 ng per sample) was amplified and labeled using the Applause WT-Amp ST and Encore Biotin Module kit (NuGEN, San Carlos, CA) and hybridized to the arrays as described by the manufacturer (Affymetrix, Santa Clara, CA). The complementary RNA hybridization cocktail was incubated overnight at 45°C while rotating in a hybridization oven. After 18 h of hybridization, the cocktail was removed and the arrays were washed and stained in an Affymetrix GeneChip fluidics station 450. Arrays were scanned on an Affymetrix GeneChip 3000G scanner. All cel files were analyzed by Bioconductor SimpleAffy to assess data quality [1]. Affymetrix data were preprocessed, normalized, summarized and annotated with the robust multi-average (RMA) method, quantile normalization and cdf file implemented in the Bioconductor affy, gcrma and annaffy packages [2].

We used the significance analysis of microarrays (SAM) to determine significant differentially expressed genes (DEGs) between sirolimus and vehicle treatment [3]. All possible permutations (seventy) were used and genes with a fold change over 1.5 and a delta value over 0.5 were assigned as DEGs resulting in a false discovery rate (median) of 10%. DEGs were hierarchically clustered and graphically represented using the MultiExperiment Viewer (MeV) developed at The Institute for Genomic Research (TIGR) [4]. The Pearson correlation and complete linkage were used as distance measure and linkage rule in the hierarchical cluster algorithm,
respectively [4, 5].

DEGs were furthermore analyzed with respect to their molecular functions, associated biological processes, and cellular locations using gene ontology terms (GO-Terms) as provided by the Gene Ontology Consortium [6]. Functional grouping of genes was based on GO-Terms and PANTHER (Protein ANalysis THrough Evolutionary Relationships) ontologies [7, 8].

Raw data files as well as the MIAME checklist are available at the GEO Omnibus Database record GSE35048.

## Results

A PDF file of tables and figures of further bioinformatical data evaluation is available at the journals website. Gene Expression Omnibus: http://www.ncbi.nlm.nih.gov/geo/query/acc.cgi?token=xdyplqkoackcgzs&acc=GSE3

5048


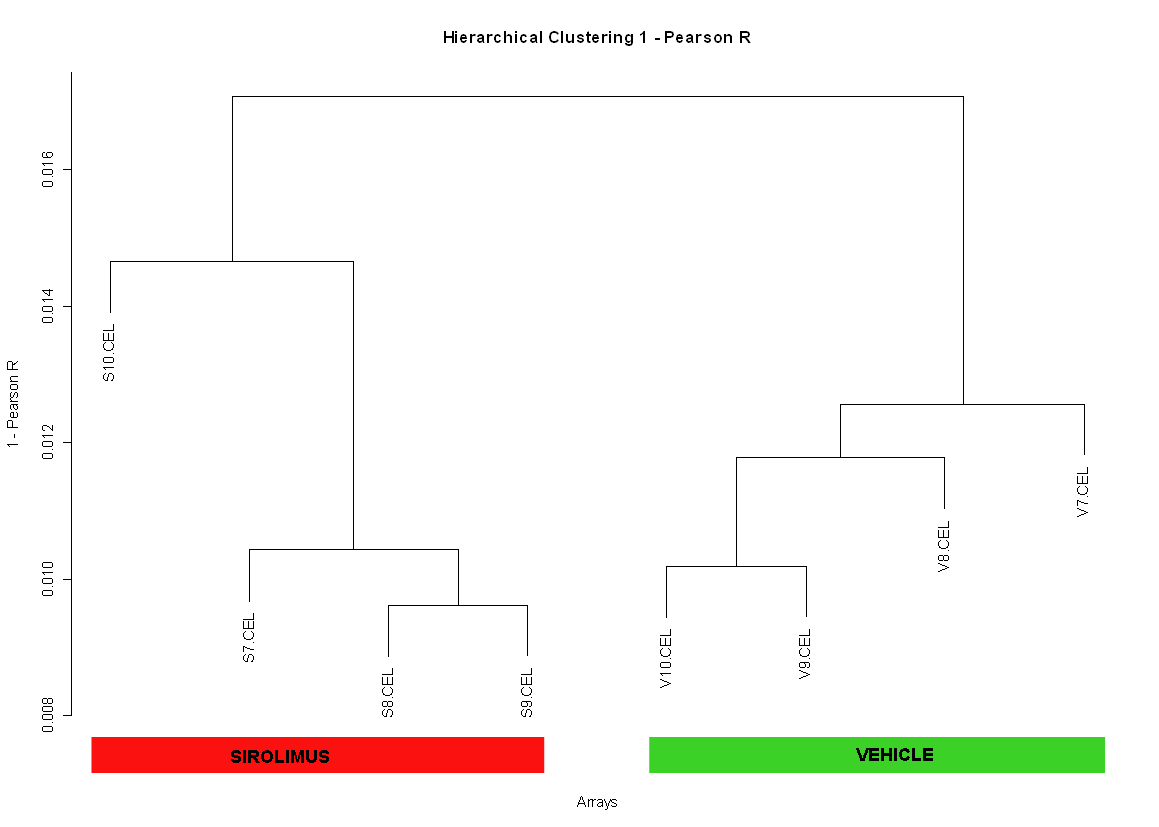


**Figure S1** Unsupervised hierarchical clustering. Unsupervised hierarchical clustering, 21131 transcripts, sirolimus treatment day 7 (pearson correlation, complete linkage)

Unsupervised hierarchical clustering of the eight samples showed a perfect separation of sirolimus treatment (figure S1).

In total 154 features were identified as significant differentially expressed when comparing the gene expression profiles of rat kidneys between sirolimus and vehicle treatment with a fold change over 1.5, resulting in 139 down-regulated and 15 up-regulated genes in the sirolimus group (figure S2, table S1).


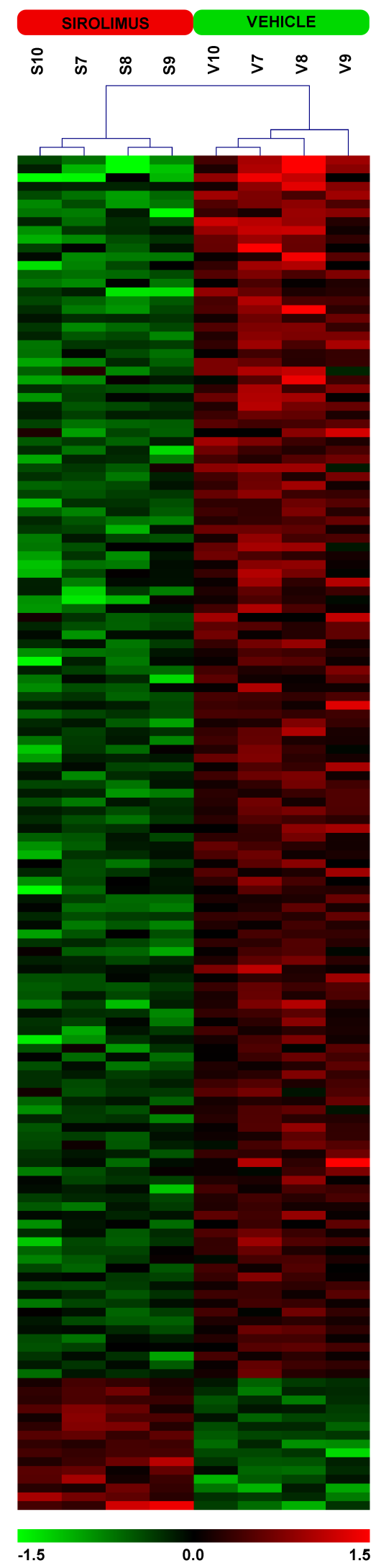


**Figure S2** Dendrogram of gene expression profiles. Dendrogram derived by unsupervised hierarchical clustering of gene expression profiles characterizing the sirolimus group (red bar) and the vehicle group (green bar). Red spots ndicate up-regulated transcripts, whereas green spots indicate down-regulated transcripts relative to the other group.

**Table S4** Significant differentially expressed genes between sirolimus (S) and vehicle (V) treatment listed by fold change.

| **Ensembl_Gene** | **Gene Symbol** | **Name** | **Mean S (n=4)** | **SEM S** | **Mean V (n=4)** | **SEM V** | **t-test** | **Fold change** |
| --- | --- | --- | --- | --- | --- | --- | --- | --- |
| ENSRNOG00000013552 | Scd1 | Stearoyl-Coenzyme A desaturase 1 | 6.74 | 0.34 | 8.88 | 0.42 | 0.007 | 0.227 |
| ENSRNOG00000012404 | Thrsp | Thyroid hormone responsive | 7.30 | 0.42 | 9.41 | 0.33 | 0.007 | 0.232 |
| ENSRNOG00000015308 | Pbk | PDZ binding kinase | 5.50 | 0.46 | 7.56 | 0.31 | 0.010 | 0.240 |
| ENSRNOG00000001796 | Dgkg | Diacylglycerol kinase, gamma | 7.57 | 0.10 | 9.00 | 0.12 | 0.000 | 0.371 |
| ENSRNOG00000015850 | Rbp7 | Retinol binding protein 7, cellular | 5.30 | 0.14 | 6.69 | 0.32 | 0.008 | 0.381 |
| ENSRNOG00000039103 |  | Transcribed locus, moderately similar to XP_002725225.1 PREDICTED: mCG140493-like [Rattus norvegicus] | 6.36 | 0.32 | 7.74 | 0.18 | 0.010 | 0.385 |
| ENSRNOG00000037198 | Usp18 | Ubiquitin specific peptidase 18 | 5.29 | 0.10 | 6.63 | 0.09 | 0.000 | 0.394 |
| ENSRNOG00000023272 | Rrm2 | Ribonucleotide reductase M2 | 6.33 | 0.17 | 7.60 | 0.13 | 0.001 | 0.417 |
| ENSRNOG00000031211 | Acsm5 | Acyl-CoA synthetase medium-chain family member 5 | 6.61 | 0.31 | 7.87 | 0.16 | 0.012 | 0.418 |
| ENSRNOG00000015550 | Ptgds | prostaglandin D2 synthase 21kDa | 7.76 | 0.10 | 9.01 | 0.22 | 0.002 | 0.421 |
| ENSRNOG00000003202 |  |  | 4.89 | 0.16 | 6.12 | 0.26 | 0.007 | 0.427 |
| ENSRNOG00000032404 | Top2a | topoisomerase (DNA) II alpha 170kDa | 7.14 | 0.28 | 8.35 | 0.25 | 0.018 | 0.433 |
| ENSRNOG00000012640 | Dpp7 | Dipeptidylpeptidase 7 | 7.26 | 0.05 | 8.45 | 0.08 | 0.000 | 0.439 |
| ENSRNOG00000037304 | Casc5 | cancer susceptibility candidate 5 | 4.88 | 0.08 | 6.05 | 0.16 | 0.001 | 0.446 |
| ENSRNOG00000026440 | RGD1559891 |  | 4.73 | 0.21 | 5.88 | 0.21 | 0.008 | 0.449 |
| ENSRNOG00000019542 | MGC108823;RGD1559715 | Similar to interferon-inducible GTPase | 4.99 | 0.19 | 6.13 | 0.14 | 0.003 | 0.453 |
| ENSRNOG00000001821 | Adipoq | Adiponectin, C1Q and collagen domain containing | 6.69 | 0.17 | 7.82 | 0.31 | 0.019 | 0.456 |
| ENSRNOG00000009086 | Apcs | Amyloid P component, serum | 9.41 | 0.11 | 10.53 | 0.11 | 0.000 | 0.460 |
| ENSRNOG00000003959 | Rgs18 | regulator of G-protein signaling 18 | 5.86 | 0.16 | 6.97 | 0.11 | 0.001 | 0.463 |
| ENSRNOG00000017539 | Mmp9 | Matrix metallopeptidase 9 | 6.24 | 0.12 | 7.35 | 0.14 | 0.001 | 0.465 |
| ENSRNOG00000028137 | RGD1566252 |  | 6.63 | 0.22 | 7.74 | 0.22 | 0.012 | 0.465 |
| ENSRNOG00000015411 | Apobec1 | Apolipoprotein B mRNA editing enzyme, catalytic polypeptide 1 | 7.46 | 0.09 | 8.55 | 0.18 | 0.002 | 0.472 |
| ENSRNOG00000000045 | Cd244 | Cd244 molecule, natural killer cell receptor 2B4 | 4.09 | 0.22 | 5.15 | 0.31 | 0.032 | 0.481 |
| ENSRNOG00000034190 | LOC678701 | Transcribed locus, moderately similar to XP_002729795.1 PREDICTED: mCG129376-like [Rattus norvegicus] | 7.34 | 0.13 | 8.39 | 0.39 | 0.042 | 0.483 |
| ENSRNOG00000014938 | Gabrb3 | Gamma-aminobutyric acid (GABA) A receptor, beta 3 | 5.76 | 0.12 | 6.81 | 0.40 | 0.047 | 0.483 |
| ENSRNOG00000018505 | Cidea | Cell death-inducing DFFA-like effector a | 7.32 | 0.25 | 8.35 | 0.31 | 0.041 | 0.490 |
| ENSRNOG00000038738 |  |  | 7.35 | 0.25 | 8.38 | 0.09 | 0.008 | 0.490 |
| ENSRNOG00000000168 | Gatm | Glycine amidinotransferase (L-arginine:glycine amidinotransferase) | 9.95 | 0.06 | 10.97 | 0.04 | 0.000 | 0.494 |
| ENSRNOG00000002925 | Tsx | Testis specific X-linked gene | 7.07 | 0.05 | 8.08 | 0.18 | 0.001 | 0.495 |
| ENSRNOG00000006731 | Spc25 | SPC25, NDC80 kinetochore complex component, homolog (S. cerevisiae) | 6.35 | 0.19 | 7.36 | 0.23 | 0.015 | 0.495 |
| ENSRNOG00000038960 | RGD1309362 | Similar to interferon-inducible GTPase | 7.57 | 0.29 | 8.58 | 0.14 | 0.020 | 0.496 |
| ENSRNOG00000015945 | Cd3g | CD3g molecule, gamma (CD3-TCR complex) | 4.86 | 0.07 | 5.86 | 0.18 | 0.002 | 0.500 |
| ENSRNOG00000028288 | Clrn3 | Clarin 3 | 6.51 | 0.19 | 7.51 | 0.09 | 0.003 | 0.501 |
| ENSRNOG00000009785 | Cdkn3 | cyclin-dependent kinase inhibitor 3 | 6.02 | 0.31 | 7.01 | 0.11 | 0.023 | 0.502 |
| ENSRNOG00000010149 | Cmc1 | COX assembly mitochondrial protein homolog (S. cerevisiae) | 7.35 | 0.20 | 8.33 | 0.09 | 0.004 | 0.508 |
| ENSRNOG00000014407 |  | Transcribed locus, moderately similar to XP_224795.4 PREDICTED: similar to C28H8.3 [Rattus norvegicus] | 5.28 | 0.09 | 6.25 | 0.18 | 0.003 | 0.512 |
| ENSRNOG00000031078 | RGD1566137 |  | 3.26 | 0.02 | 4.22 | 0.25 | 0.008 | 0.512 |
| ENSRNOG00000004601 | Hao1 | Hydroxyacid oxidase (glycolate oxidase) 1 | 5.20 | 0.20 | 6.15 | 0.13 | 0.007 | 0.516 |
| ENSRNOG00000036701 | Actg1;LOC295810 | Actin, gamma 1 | 3.85 | 0.23 | 4.80 | 0.31 | 0.050 | 0.518 |
| ENSRNOG00000026501 | Slc6a19 | Solute carrier family 6 (neutral amino acid transporter), member 19 | 8.31 | 0.12 | 9.25 | 0.08 | 0.001 | 0.519 |
| ENSRNOG00000028626 |  |  | 6.51 | 0.24 | 7.46 | 0.09 | 0.010 | 0.520 |
| ENSRNOG00000028870 | Acot1 | acyl-CoA thioesterase 1 | 8.03 | 0.11 | 8.97 | 0.11 | 0.001 | 0.522 |
| ENSRNOG00000014541 | Ddx60;LOC684383 | Transcribed locus | 5.41 | 0.20 | 6.35 | 0.11 | 0.007 | 0.523 |
| ENSRNOG00000015763 | Cml3 | Camello-like 3 | 8.40 | 0.03 | 9.34 | 0.12 | 0.000 | 0.523 |
| ENSRNOG00000013727 | Ndc80 | Transcribed locus | 5.54 | 0.15 | 6.46 | 0.26 | 0.023 | 0.526 |
| ENSRNOG00000000632 | Cdc2 | Cell division cycle 2, G1 to S and G2 to M | 4.95 | 0.11 | 5.87 | 0.19 | 0.005 | 0.526 |
| ENSRNOG00000004921 | Nusap1 | Nucleolar and spindle associated protein 1 | 6.45 | 0.32 | 7.37 | 0.14 | 0.039 | 0.529 |
| ENSRNOG00000003802 | Pttg1 | Pituitary tumor-transforming 1 | 6.50 | 0.12 | 7.41 | 0.15 | 0.003 | 0.533 |
| ENSRNOG00000037267 | Renbp | Renin binding protein | 6.48 | 0.05 | 7.39 | 0.06 | 0.000 | 0.533 |
| ENSRNOG00000026378 | Casc5 | cancer susceptibility candidate 5 | 6.20 | 0.25 | 7.10 | 0.23 | 0.038 | 0.535 |
| ENSRNOG00000038035 | Kif4 | Kinesin family member 4 | 5.92 | 0.21 | 6.82 | 0.20 | 0.022 | 0.539 |
| ENSRNOG00000032778 | Bub1 | Budding uninhibited by benzimidazoles 1 homolog (S. cerevisiae) | 5.46 | 0.17 | 6.34 | 0.26 | 0.029 | 0.542 |
| ENSRNOG00000005348 | Pamr1 | peptidase domain containing associated with muscle regeneration 1 | 7.53 | 0.27 | 8.40 | 0.13 | 0.027 | 0.544 |
| ENSRNOG00000018454 | Apoe | Apolipoprotein E | 8.02 | 0.07 | 8.90 | 0.08 | 0.000 | 0.545 |
| ENSRNOG00000013794 | Rbp1 | Retinol binding protein 1, cellular | 9.48 | 0.11 | 10.35 | 0.12 | 0.002 | 0.546 |
| ENSRNOG00000008165 | Tpx2 | TPX2, microtubule-associated, homolog (Xenopus laevis) | 6.73 | 0.15 | 7.60 | 0.07 | 0.002 | 0.550 |
| ENSRNOG00000030689 | Ms4a6b | Membrane-spanning 4-domains, subfamily A, member 6B | 6.60 | 0.16 | 7.46 | 0.24 | 0.025 | 0.550 |
| ENSRNOG00000001959 | Mx1 | Myxovirus (influenza virus) resistance 1 | 5.78 | 0.13 | 6.63 | 0.18 | 0.008 | 0.556 |
| ENSRNOG00000000925 | Psph | Phosphoserine phosphatase | 7.75 | 0.10 | 8.59 | 0.12 | 0.002 | 0.561 |
| ENSRNOG00000013057 | Prc1 | Protein regulator of cytokinesis 1 | 6.49 | 0.26 | 7.32 | 0.16 | 0.034 | 0.562 |
| ENSRNOG00000018503 | Bcl2l1;LOC293190;LOC684140 |  | 6.16 | 0.34 | 6.99 | 0.10 | 0.061 | 0.565 |
| ENSRNOG00000000903 | Asl |  | 8.49 | 0.06 | 9.32 | 0.03 | 0.000 | 0.565 |
| ENSRNOG00000009597 | Cyp4a10;Cyp4a1 | Cytochrome P450, family 4, subfamily a, polypeptide 1 | 8.61 | 0.15 | 9.43 | 0.31 | 0.052 | 0.566 |
| ENSRNOG00000010252 | Hexa | Hexosaminidase A | 9.24 | 0.06 | 10.06 | 0.03 | 0.000 | 0.566 |
| ENSRNOG00000021735 | Akr1cl1 | Aldo-keto reductase family 1, member C-like 1 | 5.54 | 0.18 | 6.36 | 0.14 | 0.012 | 0.568 |
| ENSRNOG00000016561 | Ns5atp9 | NS5A (hepatitis C virus) transactivated protein 9 | 6.72 | 0.15 | 7.53 | 0.10 | 0.004 | 0.571 |
| ENSRNOG00000007314 | Slc26a4 | Solute carrier family 26, member 4 | 8.49 | 0.18 | 9.30 | 0.07 | 0.006 | 0.572 |
| ENSRNOG00000039098 | Vom2r64 | Vomeronasal 2 receptor, 64 | 4.84 | 0.19 | 5.64 | 0.15 | 0.015 | 0.574 |
| ENSRNOG00000020480 | Fads1 | Fatty acid desaturase 1 | 8.52 | 0.16 | 9.31 | 0.14 | 0.010 | 0.578 |
| ENSRNOG00000005556 | Snrpf | Small nuclear ribonucleoprotein polypeptide F | 7.05 | 0.16 | 7.83 | 0.24 | 0.035 | 0.581 |
| ENSRNOG00000029369 | Olr1673 | Olfactory receptor 1673 | 3.64 | 0.12 | 4.42 | 0.12 | 0.003 | 0.583 |
| ENSRNOG00000031335 | Ankrd37 | Ankyrin repeat domain 37 | 7.77 | 0.13 | 8.54 | 0.12 | 0.005 | 0.585 |
| ENSRNOG00000026605 | Ifi27l2b |  | 7.51 | 0.09 | 8.28 | 0.06 | 0.000 | 0.586 |
| ENSRNOG00000037626 |  |  | 6.23 | 0.22 | 6.99 | 0.13 | 0.023 | 0.587 |
| ENSRNOG00000038407 |  |  | 3.01 | 0.05 | 3.78 | 0.19 | 0.008 | 0.588 |
| ENSRNOG00000010721 | Dlgap5 | discs, large (Drosophila) homolog-associated protein 5 | 6.19 | 0.17 | 6.96 | 0.10 | 0.009 | 0.589 |
| ENSRNOG00000002711 | Nuf2 | NUF2, NDC80 kinetochore complex component, homolog (S. cerevisiae) | 5.74 | 0.06 | 6.50 | 0.12 | 0.001 | 0.591 |
| ENSRNOG00000009513 | Akr1b1;Akr1b1-ps2 | Aldo-keto reductase family 1, member B1 (aldose reductase) | 9.92 | 0.09 | 10.68 | 0.20 | 0.013 | 0.593 |
| ENSRNOG00000007139 | Ttpa | Tocopherol (alpha) transfer protein | 6.49 | 0.10 | 7.24 | 0.12 | 0.003 | 0.596 |
| ENSRNOG00000038686 | Ap1s2 | Adaptor-related protein complex 1, sigma 2 subunit | 6.17 | 0.19 | 6.92 | 0.09 | 0.013 | 0.598 |
| ENSRNOG00000032417 | Gabrp | gamma-aminobutyric acid (GABA) A receptor, pi | 8.45 | 0.07 | 9.19 | 0.27 | 0.037 | 0.598 |
| ENSRNOG00000011598 | Slc15a1 | Solute carrier family 15 (oligopeptide transporter), member 1 | 7.14 | 0.22 | 7.88 | 0.13 | 0.027 | 0.598 |
| ENSRNOG00000003927 | Cd55 | Transcribed locus | 6.86 | 0.16 | 7.60 | 0.07 | 0.006 | 0.600 |
| ENSRNOG00000005037 | Kif18a | Kinesin family member 18A | 6.62 | 0.19 | 7.36 | 0.17 | 0.028 | 0.600 |
| ENSRNOG00000038792 | Olr1662;LOC682056 | Olfactory receptor 1662 | 4.03 | 0.16 | 4.76 | 0.12 | 0.010 | 0.602 |
| ENSRNOG00000028185 | LOC500420 | Similar to CG12279-PA | 7.41 | 0.18 | 8.14 | 0.10 | 0.012 | 0.602 |
| ENSRNOG00000005180 |  |  | 6.02 | 0.12 | 6.75 | 0.11 | 0.004 | 0.605 |
| ENSRNOG00000021357 | Slfn3 | Schlafen 3 | 6.75 | 0.15 | 7.47 | 0.20 | 0.026 | 0.607 |
| ENSRNOG00000010283 | Cd28 | Cd28 molecule | 5.68 | 0.08 | 6.40 | 0.23 | 0.025 | 0.608 |
| ENSRNOG00000001926 | Cldn1 | Claudin 1 | 8.59 | 0.09 | 9.31 | 0.06 | 0.000 | 0.609 |
| ENSRNOG00000015857 | Ctsa | Cathepsin A | 11.03 | 0.05 | 11.74 | 0.08 | 0.000 | 0.611 |
| ENSRNOG00000022505 | Slc17a4;LOC684568 | Solute carrier family 17 (urate transporter), member 4 | 7.96 | 0.08 | 8.66 | 0.19 | 0.015 | 0.614 |
| ENSRNOG00000006395 | RGD1561343 | Similar to C20orf118 | 6.16 | 0.12 | 6.87 | 0.06 | 0.002 | 0.614 |
| ENSRNOG00000039801 | RGD1563091 |  | 6.72 | 0.15 | 7.42 | 0.08 | 0.006 | 0.615 |
| ENSRNOG00000030250 |  |  | 3.92 | 0.15 | 4.62 | 0.09 | 0.007 | 0.617 |
| ENSRNOG00000004405 | Pigr | Polymeric immunoglobulin receptor | 8.56 | 0.17 | 9.25 | 0.12 | 0.017 | 0.622 |
| ENSRNOG00000001333 | Azgp1 | alpha-2-glycoprotein 1, zinc-binding | 8.25 | 0.26 | 8.94 | 0.08 | 0.048 | 0.622 |
| ENSRNOG00000001295 | S100b | Transcribed locus | 5.93 | 0.05 | 6.61 | 0.12 | 0.002 | 0.622 |
| ENSRNOG00000012804 | C1qc | Complement component 1, q subcomponent, C chain | 7.27 | 0.08 | 7.95 | 0.11 | 0.002 | 0.624 |
| ENSRNOG00000023093 | LOC689296 | Similar to expressed sequence C79407 | 5.34 | 0.09 | 6.02 | 0.12 | 0.004 | 0.627 |
| ENSRNOG00000018812 | Rpp25 | Ribonuclease P 25 subunit (human) | 7.66 | 0.13 | 8.33 | 0.10 | 0.006 | 0.628 |
| ENSRNOG00000011622 | Echdc1 | Enoyl Coenzyme A hydratase domain containing 1 | 6.63 | 0.14 | 7.30 | 0.17 | 0.024 | 0.629 |
| ENSRNOG00000014224 | LOC684509;Ndufa3 |  | 8.61 | 0.20 | 9.28 | 0.06 | 0.017 | 0.629 |
| ENSRNOG00000038572 | RGD1562646 | Similar to chromosome condensation protein G | 5.51 | 0.05 | 6.17 | 0.11 | 0.001 | 0.631 |
| ENSRNOG00000029924 | Klk1l | Kallikrein 1-like peptidase | 10.77 | 0.11 | 11.43 | 0.20 | 0.026 | 0.632 |
| ENSRNOG00000003388 | Cenpf | centromere protein F, 350/400kDa (mitosin) | 6.78 | 0.20 | 7.44 | 0.15 | 0.038 | 0.632 |
| ENSRNOG00000033996 | RGD1565900 | Transcribed locus, strongly similar to NP_990668.1 60S ribosomal protein L27 [Gallus gallus] | 9.71 | 0.20 | 10.37 | 0.14 | 0.037 | 0.634 |
| ENSRNOG00000004280 | Tcn2 | Transcobalamin 2 | 8.91 | 0.05 | 9.56 | 0.07 | 0.000 | 0.635 |
| ENSRNOG00000029778 | Maob | Monoamine oxidase B | 6.32 | 0.04 | 6.97 | 0.13 | 0.003 | 0.635 |
| ENSRNOG00000022657 | Tmem97 | Transmembrane protein 97 | 7.23 | 0.12 | 7.89 | 0.11 | 0.007 | 0.637 |
| ENSRNOG00000019716 | Ntf3 | Transcribed locus | 7.19 | 0.04 | 7.84 | 0.13 | 0.004 | 0.640 |
| ENSRNOG00000029370 | Abhd3 |  | 7.39 | 0.20 | 8.04 | 0.13 | 0.037 | 0.641 |
| ENSRNOG00000039696 | Olr1547 | Olfactory receptor 1547 | 2.70 | 0.13 | 3.34 | 0.17 | 0.024 | 0.642 |
| ENSRNOG00000002382 | Mfap4 | Microfibrillar-associated protein 4 | 7.16 | 0.20 | 7.80 | 0.14 | 0.043 | 0.643 |
| ENSRNOG00000029055 | Ttk | Ttk protein kinase | 4.92 | 0.10 | 5.56 | 0.10 | 0.004 | 0.643 |
| ENSRNOG00000024159 | Fcer1g | Fc fragment of IgE, high affinity I, receptor for; gamma polypeptide | 9.97 | 0.18 | 10.61 | 0.12 | 0.024 | 0.644 |
| ENSRNOG00000017332 | Dapk2 | Death-associated kinase 2 | 6.72 | 0.02 | 7.35 | 0.25 | 0.043 | 0.645 |
| ENSRNOG00000007089 | Lgmn | Legumain | 10.90 | 0.12 | 11.53 | 0.05 | 0.003 | 0.646 |
| ENSRNOG00000028746 | Gsto1 | Glutathione S-transferase omega 1 | 9.08 | 0.08 | 9.71 | 0.11 | 0.004 | 0.646 |
| ENSRNOG00000029057 | Olr898 | Olfactory receptor 898 | 4.28 | 0.16 | 4.91 | 0.17 | 0.039 | 0.647 |
| ENSRNOG00000004667 | Gen1 | Gen homolog 1, endonuclease (Drosophila) | 5.23 | 0.14 | 5.85 | 0.17 | 0.026 | 0.647 |
| ENSRNOG00000019662 | Tm6sf1 | Transmembrane 6 superfamily member 1 | 8.31 | 0.05 | 8.94 | 0.05 | 0.000 | 0.648 |
| ENSRNOG00000013215 | Dctd | dCMP deaminase | 7.11 | 0.19 | 7.73 | 0.08 | 0.025 | 0.651 |
| ENSRNOG00000000137 | Ly86 | Lymphocyte antigen 86 | 7.50 | 0.11 | 8.12 | 0.17 | 0.022 | 0.651 |
| ENSRNOG00000002253 | Wdr5b | WD repeat domain 5B | 5.91 | 0.13 | 6.53 | 0.12 | 0.014 | 0.651 |
| ENSRNOG00000017259 | Tacc3 | Transforming, acidic coiled-coil containing protein 3 | 6.15 | 0.13 | 6.77 | 0.08 | 0.006 | 0.652 |
| ENSRNOG00000018815 | Plk1 | Polo-like kinase 1 (Drosophila) | 6.28 | 0.13 | 6.90 | 0.13 | 0.015 | 0.653 |
| ENSRNOG00000000853 | Aif1 | Allograft inflammatory factor 1 | 8.36 | 0.08 | 8.97 | 0.06 | 0.001 | 0.653 |
| ENSRNOG00000018899 | C5 | Complement component 5 | 8.74 | 0.09 | 9.35 | 0.09 | 0.003 | 0.655 |
| ENSRNOG00000019189 | Acat2;RGD1561787 | Transcribed locus | 9.78 | 0.14 | 10.38 | 0.08 | 0.010 | 0.656 |
| ENSRNOG00000014064 | Ctsh | Cathepsin H | 11.50 | 0.10 | 12.11 | 0.03 | 0.001 | 0.656 |
| ENSRNOG00000036674 | Cd7 | Cd7 molecule | 6.76 | 0.14 | 7.36 | 0.14 | 0.024 | 0.660 |
| ENSRNOG00000037940 |  |  | 4.83 | 0.08 | 5.43 | 0.18 | 0.025 | 0.660 |
| ENSRNOG00000016717 | Gas2 | Growth arrest-specific 2 | 7.38 | 0.09 | 7.97 | 0.16 | 0.016 | 0.662 |
| ENSRNOG00000006859 | Insig1 | insulin induced gene 1 | 9.09 | 0.12 | 9.68 | 0.13 | 0.014 | 0.663 |
| ENSRNOG00000036402 |  |  | 5.03 | 0.03 | 5.62 | 0.17 | 0.015 | 0.664 |
| ENSRNOG00000028623 | Agpat5 | Transcribed locus | 5.88 | 0.11 | 6.47 | 0.11 | 0.008 | 0.665 |
| ENSRNOG00000013001 |  |  | 6.42 | 0.12 | 7.01 | 0.12 | 0.013 | 0.666 |
| ENSRNOG00000033056 |  |  | 6.17 | 0.09 | 6.76 | 0.13 | 0.010 | 0.666 |
| ENSRNOG00000038319 | Akr1c19 | Transcribed locus | 9.70 | 0.06 | 9.10 | 0.09 | 0.002 | 1.512 |
| ENSRNOG00000001809 | Hrg;LOC681544 | Histidine-rich glycoprotein | 9.17 | 0.10 | 8.40 | 0.12 | 0.003 | 1.701 |
| ENSRNOG00000007923 | Cgref1 | Cell growth regulator with EF hand domain 1 | 8.75 | 0.14 | 7.99 | 0.07 | 0.002 | 1.701 |
| ENSRNOG00000033527 | Pappa |  | 7.95 | 0.04 | 7.16 | 0.11 | 0.001 | 1.721 |
| ENSRNOG00000011184 | Slc13a4 | Solute carrier family 13 (sodium/sulfate symporters), member 4 | 7.15 | 0.14 | 6.32 | 0.10 | 0.003 | 1.780 |
| ENSRNOG00000023465 | LOC500300 | Similar to hypothetical protein MGC6835 | 7.10 | 0.13 | 6.26 | 0.14 | 0.004 | 1.785 |
| ENSRNOG00000018693 | Asgr1 | Asialoglycoprotein receptor 1 | 7.44 | 0.15 | 6.57 | 0.09 | 0.002 | 1.835 |
| ENSRNOG00000017944 | Tbx10 | Transcribed locus | 7.35 | 0.06 | 6.45 | 0.05 | 0.000 | 1.876 |
| ENSRNOG00000019260 | Ceacam20 |  | 8.30 | 0.20 | 7.35 | 0.07 | 0.004 | 1.930 |
| ENSRNOG00000036754 |  |  | 9.59 | 0.04 | 8.63 | 0.14 | 0.001 | 1.939 |
| ENSRNOG00000028616 | Pck1 | Phosphoenolpyruvate carboxykinase 1 (soluble) | 11.53 | 0.02 | 10.53 | 0.22 | 0.004 | 2.009 |
| ENSRNOG00000015992 | Ccl20 | Chemokine (C-C motif) ligand 20 | 8.03 | 0.16 | 7.01 | 0.11 | 0.002 | 2.026 |
| ENSRNOG00000029066 |  |  | 7.30 | 0.18 | 6.27 | 0.19 | 0.008 | 2.035 |
| ENSRNOG00000006367 | Slc5a8 | Solute carrier family 5 (iodide transporter), member 8 | 9.46 | 0.11 | 8.40 | 0.21 | 0.004 | 2.086 |
| ENSRNOG00000031231 | Kng1;Kng2 | Kininogen 1 | 7.27 | 0.28 | 5.85 | 0.17 | 0.005 | 2.681 |

**Table S5** Functional classification of DEGs using PANTHER ontologies. Enriched biological processes separating sirolimus from vehicle group as derived on the level of differential gene expression by SAM. Categories are ranked by the p-value (comparison of expected number of genes and observed number of genes in each biological process) indicating the relevance of a particular process.

| **Enriched Biological Processes** | | |
| --- | --- | --- |
| **down-regulated genes by Sirolimus treatment** | | |
| **biological process** | **number of genes (n=76)** | **p-value** |
| response to stimulus | 21 | <0.001 |
| immune system process | 23 | <0.001 |
| lipid metabolic process | 13 | <0.001 |
| immune response | 11 | <0.001 |
| cellular process | 35 | <0.001 |
| anion transport | 4 | 0.001 |
| cell cycle | 14 | 0.003 |
| Transport | 18 | 0.003 |
| B cell mediated immunity | 6 | 0.004 |
| Mitosis | 7 | 0.005 |
| cellular amino acid and derivative metabolic process | 5 | 0.006 |
| metabolic process | 40 | 0.008 |
| cellular defense response | 6 | 0.009 |
| cellular component morphogenesis | 9 | 0.014 |
| anatomical structure morphogenesis | 9 | 0.014 |
| complement activation | 3 | 0.015 |
| signal transduction | 22 | 0.016 |
| developmental process | 17 | 0.017 |
| primary metabolic process | 37 | 0.020 |
| cell communication | 22 | 0.024 |
| vitamin metabolic process | 2 | 0.027 |
| induction of apoptosis | 4 | 0.027 |
| ion transport | 6 | 0.028 |
| defense response to bacterium | 2 | 0.028 |
| vitamin transport | 2 | 0.035 |
| coenzyme metabolic process | 2 | 0.036 |
| Cytokinesis | 3 | 0.039 |
| cell-cell signaling | 8 | 0.040 |
| chromosome segregation | 3 | 0.042 |
| **up-regulated genes by Sirolimus treatment** | | |
| **biological process** | **number of genes (n=8)** | **p-value** |
| response to external stimulus | 3 | <0.001 |
| blood coagulation | 3 | <0.001 |
| response to stimulus | 4 | 0.005 |
| immune system process | 4 | 0.011 |
| regulation of biological process | 1 | 0.020 |
| regulation of vasoconstriction | 1 | 0.020 |
| anion transport | 1 | 0.046 |

According to PANTHER classification down-regulated transcripts belong to response to stimulus, metabolic processes, immune system, transport and signal transduction. Up-regulated transcripts belong also to response to stimulus, immune system and transport but also to blood coagulation and regulation of vasoconstriction (table S2).

**Table S6** Na+/K+ -ATPase subunits expression levels between sirolimus and vehicle group measured on the GeneChip expression array. None of the polypeptides forming Na+/K+ -ATPases are differentially expressed between the groups.

| Gene | Ensembl_Gene | EntrezGene | Description | mean S (n=4) | mean V (n=4) | fold change | p-value |
| --- | --- | --- | --- | --- | --- | --- | --- |
| Atp1a2 | ENSRNOG00000007290 | 24212 | ATPase, Na+/K+ transporting, alpha 2 polypeptide | 6.49 | 6.98 | 1.40 | 0.077 |
| Atp1a3 | ENSRNOG00000020263 | 24213 | ATPase, Na+/K+ transporting, alpha 3 polypeptide | 5.98 | 5.97 | 1.00 | 0.972 |
| Atp1b4 | ENSRNOG00000007059 | 84396 | ATPase, (Na+)/K+ transporting, beta 4 polypeptide | 5.11 | 5.25 | 1.10 | 0.296 |
| Atp1b2 | ENSRNOG00000011227 |  | ATPase, Na+/K+ transporting, beta 2 polypeptide | 7.25 | 7.17 | 1.06 | 0.727 |
| Atp1b3 | ENSRNOG00000011501 | 25390 | ATPase, Na+/K+ transporting, beta 3 polypeptide | 9.53 | 9.67 | 1.11 | 0.287 |

## References

1. Wilson, Miller. Simpleaffy: a BioConductor package for Affymetrix quality control and data analysis. *Bioinformatics*. 2005.

2. Irizarry RA, Hobbs B, Collin F, et al. Exploration, normalization, and summaries of high density oligonucleotide array probe level data. *Biostatistics*. 2003; **4**: 249-64.

3. Tusher VG, Tibshirani R, Chu G. Significance analysis of microarrays applied to the ionizing radiation response. *Proc Natl Acad Sci U S A*. 2001; **98**: 5116-21.

4. Saeed AI, Sharov V, White J, et al. TM4: a free, open-source system for microarray data management and analysis. *Biotechniques*. 2003; **34**: 374-8.

5. Eisen MB, Spellman PT, Brown PO, Botstein D. Cluster analysis and display of genome-wide expression patterns. *Proc Natl Acad Sci U S A*. 1998; **95**: 14863-8.

6. Ashburner M, Ball CA, Blake JA, et al. Gene ontology: tool for the unification of biology. The Gene Ontology Consortium. *Nat Genet*. 2000; **25**: 25-9.

7. Mi H, Lazareva-Ulitsky B, Loo R, et al. The PANTHER database of protein families, subfamilies, functions and pathways. *Nucleic Acids Res*. 2005; **33**: D284-8.

8. Hoffmann R, Valencia A. A gene network for navigating the literature. *Nat Genet*. 2004; **36**: 664.
